# Our Forthcoming Series of Articles

**Published:** 1916-04

**Authors:** 


### Our Forthcoming Series of Articles.

The interest being manifested in our announcement last month (p. 366) that we purposed publishing a series of practical articles by Dr. Henry R. Harrower on *"The Diagnosis of the Internal Secretory Disorders,"* to start in our May issue, is as encouraging as it is surprising. Evidently the profession is very much awake to the growing importance of the glands of internal secretion in every-day practice.

The manuscript for several of the papers is already in hand, and some of it is in type. It is excellent, and we are enthusiastic about it. The subjects are interesting and practical, the writer's style is pleasing, and between the lines we can see an enthusiasm which we are sure will infect those who "read, mark and inwardly digest" Dr. Harrower's articles.

The title of the first paper which will appear in our next issue is "The Frequency of Internal Secretory Disorders in Every-day Practice," and it emphasizes the insidiousness of these disturbances and their intimacy with many well-known conditions. As Dr. Harrower says: "Too often we are prone to look upon this class of cases as rare and occasional, and their diagnosis as comparatively easy because of the obviousness of such diseases as cretinism, myxedema, giantism or acromegaly. This is a mistake, for functional disorders of this class are of every-day occurrence and are far more important than the more obvious organic diseases, for they are still in their early stages before serious disharmony is caused and are naturally more responsive to suitable treatment."

The next two papers (for June and July) take up the disorders of the thyroid apparatus, the first one being "The Detection of the Minor Thyroid Dyscrasias," and the second, "The More Serious Organic Thyroid Diseases." These are important papers, for when the thyroid is better understood it will be surprising how many times it enters into and complicates the commonest clinical disorders.

You cannot afford to miss one article in this excellent series.

